# Atrophy and Primary Somatosensory Cortical Reorganization after Unilateral Thoracic Spinal Cord Injury: A Longitudinal Functional Magnetic Resonance Imaging Study

**DOI:** 10.1155/2013/753061

**Published:** 2013-12-29

**Authors:** Jia-Sheng Rao, Ma Manxiu, Can Zhao, Yue Xi, Zhao-Yang Yang, Liu Zuxiang, Xiao-Guang Li

**Affiliations:** ^1^Department of Biomedical Engineering, School of Biological Science and Medical Engineering, Beihang University, Beijing 100191, China; ^2^State Key Laboratory of Brain and Cognitive Science, Institute of Biophysics, Chinese Academy of Sciences, Beijing 100101, China; ^3^Beijing Institute for Neuroscience, Capital Medical University, Beijing 100069, China

## Abstract

The effects of traumatic spinal cord injury (SCI) on the changes in the central nervous system (CNS) over time may depend on the dynamic interaction between the structural integrity of the spinal cord and the capacity of the brain plasticity. Functional magnetic resonance imaging (fMRI) was used in a longitudinal study on five rhesus monkeys to observe cerebral activation during upper limb somatosensory tasks in healthy animals and after unilateral thoracic SCI. The changes in the spinal cord diameters were measured, and the correlations among time after the lesion, structural changes in the spinal cord, and primary somatosensory cortex (S1) reorganization were also determined. After SCI, activation of the upper limb in S1 shifted to the region which generally dominates the lower limb, and the rostral spinal cord transverse diameter adjacent to the lesion exhibited obvious atrophy, which reflects the SCI-induced changes in the CNS. A significant correlation was found among the time after the lesion, the spinal cord atrophy, and the degree of contralateral S1 reorganization. The results indicate the structural changes in the spinal cord and the dynamic reorganization of the cerebral activation following early SCI stage, which may help to further understand the neural plasticity in the CNS.

## 1. Introduction

Spinal cord injury (SCI) disrupts the ascending sensory nerve fiber tract and the descending motor nerve fiber tract, thereby triggering the loss of somatosensory afferent information and motor control of all body parts below the level of the lesion in the corresponding cerebral cortex. The axonal disintegration and path destruction caused by SCI regulates the flow of incoming and outgoing information between the brain and the spinal cord and leads to reorganization and changes in residual neural circuits [1]. Studies have shown that incomplete SCI patients exhibit more active sensorimotor and premotor cortices during motor tasks [2–7]. The loss of afferent sensory information has been confirmed to generate the functional reorganization of the primary somatosensory cortex (S1) [8–12]. The significance of cortical reorganization after SCI during functional recovery is poorly understood. Moreover, SCI patients often suffer from spinal cord atrophy [13]. Freund et al. [14] carried out contrast enhanced MRI experiments on the upper limb of 10 patients with chronic cervical cord injury and healthy subjects to verify cervical cord atrophy and its correlation with the degree of cortical reorganization. Lundell et al. [7] investigated the changes in cerebral activation in 19 chronic SCI patients during lower limb movements and found that cortical reorganization is negatively correlated with the transverse diameter (TD) of the spinal cord but independent of the anteroposterior diameter (AD). These studies revealed the correlation between the significant cortical reorganization of chronic SCI and spinal cord atrophy.

SCI triggers a series of pathophysiologic events, including initial cell death, hemorrhage, and further tissue damage [15]. In acute SCI, treatment of the injured region can protect the remaining neurons [16] and partly restore nerve function [17]. Previous studies have reported cortical reorganization during the early stages of SCI in rats [18, 19]. However, the process of the S1 reorganization during the early stage of thoracic spinal cord injury in primates has not been reported. Correlations among time points after the lesion, structural changes of the spinal cord, and S1 cortical reorganization are still unclear. We investigated the early atrophic changes during SCI and cortical reorganization to further understand the plasticity of the central nervous system, as well as assessing the potential for clinical treatment. We used blood oxygenation level-dependent functional magnetic resonance imaging (BOLD-fMRI) to record cerebral activation of temperature-stimulated upper limbs contralateral to the injury in five female rhesus monkeys at healthy stage and then at 4, 8, and 12 weeks after unilateral thoracic SCI. We also measured the spinal cord AD and TD at the caudal and rostral borders of the lesion and the second segment of the cervical cord. Then, we assessed the longitudinal changes in cerebral activation and spinal cord atrophy at each time point and analyzed the correlations among the time points after the lesion, the degree of cortical reorganization, and the changes in the spinal cord structure. We also determined whether trauma-induced spinal cord changes are correlated with task-induced cerebral activation.

## 2. Methods

### 2.1. Animal Preparation and Stimulation Protocol

Five adult female rhesus monkeys (weight: 5 kg ± 1 kg; age: 6 years ± 1 year old) were used in our animal experiment. The study was approved by the Animal Ethics Committee of Capital Medical University. SCI was conducted via hemisection of section T7–T9 on the right side of the spinal cord (10 mm in length and 3 mm in width). fMRI and imaging of the corresponding anatomical brain structures were conducted before and after the surgery. The scanning time points were the preoperative period and then at 4, 8, and 12 weeks after the operation. The time points were selected based on the premise of the steady state of the SCI animals. Prior to scanning, each monkey was anesthetized via an intramuscular injection of ketamine hydrochloride solution (10 mg/kg) and atropine sulfate injection (0.05 mg/kg) to decrease bronchial and salivary secretions. Then, anesthesia was maintained during the scan via constant intravenous propofol (0.25 mg/kg/min) [20, 21]. During scanning, the anesthesia level was monitored at regular intervals, with the following reactions as the standard: no somatic movement when toes are pinched, loss of corneal reflex with a sustained heart rate of at least 70 times per minute, and respiration rate higher than 20 times per minute [22]. For the somatosensory test, a domestic ice-water mixture container was used for innocuous cold stimulation (6°C). The medial cutaneous surface of the gastrocnemius (left lower limb) and the medial cutaneous surface of the carpal area (left upper limb) contralateral to the SCI were stimulated. The block design was adopted with a 20 s stimulation period, followed by a 20 s rest period. The stimulation blocks and the rest blocks were alternated and were repeated 4 times. During the first stimulation period, an extra 20 s was added to obtain the baseline hemodynamic response. The stimulated regions were arranged in a pseudorandomized order. Between different stimulation regions, a 3 min rest interval was applied to allow the hemodynamic response to return to the baseline.

### 2.2. MRI Acquisition

All MRI research was accomplished with the Siemens Tim 3.0T system (Siemens Medical Solutions, Erlangen, Germany). Structural and functional images were acquired with a custom-made primate four-channel transmitter and receiver coil. The BOLD signals were obtained with the gradient echo-echo planar imaging sequence (GE-EPI) and set as follows: TR = 2,000 ms, TE = 30 ms, matrix = 64 × 64, field of view (FOV) = 128 mm × 128 mm, flip angle = 90°, 25 consecutive slices of the axial image covered the entire brain, and voxel spatial resolution was 2 mm × 2 mm × 2 mm. Before each functional imaging scan, 4 s of empty scanning was adopted to avoid the magnetic field heterogeneity at the beginning of the scanning. Each scanning period lasted 3 min 4 s, and 90 volumes of EPI data were acquired.

The 3D magnetization prepared rapid acquisition gradient echo (MPRAGE) sequence was used to obtain high-resolution anatomical structure images, with the following parameters: TR = 1520 ms, TE = 4.42 ms, flip angle = 15°, and TI = 520 ms, the same centering to functional data, and 180 contiguous slices covering the entire brain; the voxel spatial resolution was 1.0 mm × 0.5 mm × 0.5 mm.

Anatomical images of the spinal cord were acquired using a double coil configuration with the body volume coil used as transmitter and the spine surface coil as receiver. The proton density (PD) weighted images were obtained with a turbo spin-echo (TSE) sequence. Five echoes were acquired. The imaging parameters were as follows: TR = 3050 ms, TE = 11 ms, flip angle = 149°, matrix = 320 × 320, and 27 consecutive slices of axial images covering the SCI region. The voxel spatial resolution was 0.6 mm × 0.6 mm × 2 mm. The saturated band was set in the chest and abdominal cavity to reduce physiologic motion artifacts [23].

### 2.3. Data Processing

All fMRI data were processed with SPM 8 (http://www.fil.ion.ucl.ac.uk/spm/). The first three volumes of every scan were excluded to prevent susceptibility artifacts. For the remaining images, the middle slice of each volume was used as the reference for rearrangement to fix the acquisition time delay. Then, the rigid transformation of six parameters registered all data on the first image to fix motion artifacts [24]. After motion correction, the data was registered in accordance with the anatomical structure images of each monkey and then to the standard MRI brain atlas of monkeys [25, 26]. Finally, a 3 mm isotropic Gaussian filter was used for image smoothing. We built up the activated regression analysis through the convolution block design paradigm with canonical hemodynamic response function. The activation map was generated with the SPM general linear model (GLM), with a threshold of *P* < 0.1 (FDR corrected). The low-frequency signal drift was removed with a high-pass filter at 1/100 Hz [27].

The region of interest (ROI) was positioned at the contralateral S1 (c-S1) and ipsilateral S1 (i-S1). The ROIs were manually selected according to the rhesus macaque atlas [28]. We set the intersection of the central sulcus and the dorsal longitudinal fissure as the anatomical landmark based on the methods by Wrigley et al. [29]. We acquired the Montreal Neurological Institute (MNI) coordinates of the maximum activated voxel in the posterior central gyrus of all monkeys and calculated the 3D spatial distance (SD) and spatial angle (SA) between the anatomical landmark and the maximum activated voxel. The caudal and the rostral aspects of the SCI region were positioned 2 cm from the lesion. The central level of the second segment of the cervical cord was extracted from the scans of the head structure. Based on this landmark, the spinal cord AD and TD were measured.

## 3. Statistical Analysis

Multiple comparisons were conducted using one-way ANOVA to analyze the differences in spinal cord diameters (AD and TD), S1 activation centric coordinates, and S1-activated SD and SA at four time points. A one-tailed Spearman's correlation analysis was conducted to determine significant differences in the correlation of time points after the lesion, spinal cord diameter, S1 activation center, and S1-activated SD and SA. *P* < 0.05 was considered statistically significant. The statistical analysis was conducted with SPSS version 17.0 (SPSS Inc., Chicago, IL), and all quantitative data was expressed in the form of means ± standard deviation.

## 4. Results

### 4.1. Measurement of Spinal Cord Diameter

**Figure 1:**
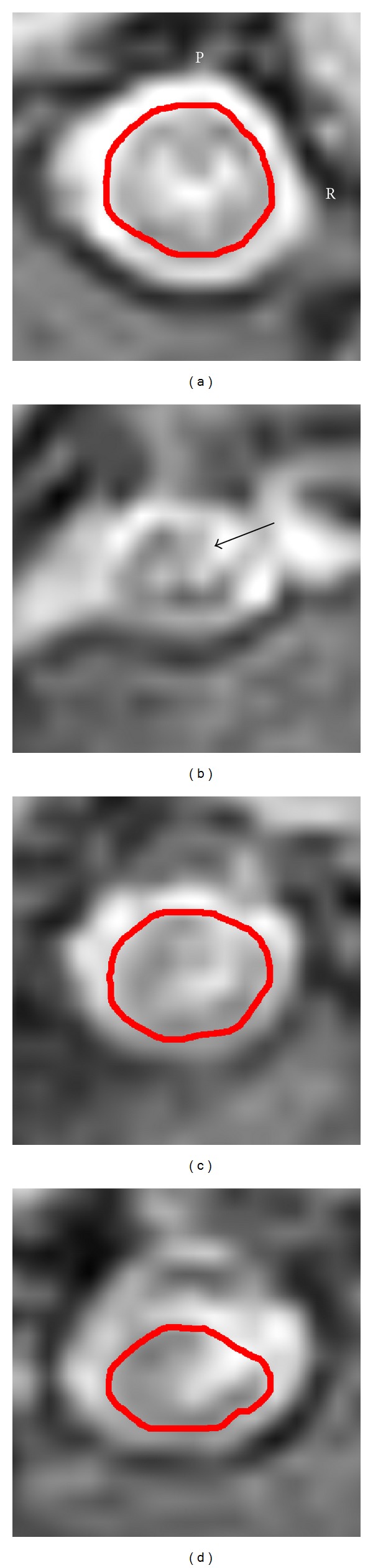
Morphological diagram of the spinal cord of healthy monkeys at 12 weeks after SCI. (a) Preoperative MRI structural image of the injured region. (b) Structural image at 12 weeks after SCI; the black arrow indicates the injured area. ((c) and (d)) Morphologies of the rostral spinal cord 2 cm from the lesion and the caudal spinal cord 2 cm from the lesion. P indicates the posterior part and R represents the right side.

The morphology of the monkey spinal cord preoperatively and after the lesion was imaged through magnetic resonance scanning (Figure 1), and the spinal cord TD and AD (the caudal and rostral views of the injured region and cervical cord) were also assessed, as shown in Table 1. The rostral TD value was significantly smaller at 12 weeks after SCI than that in the healthy stage (*P* = 0.031). whereas data at other locations showed no statistical difference at all time points.

**Figure 2:**
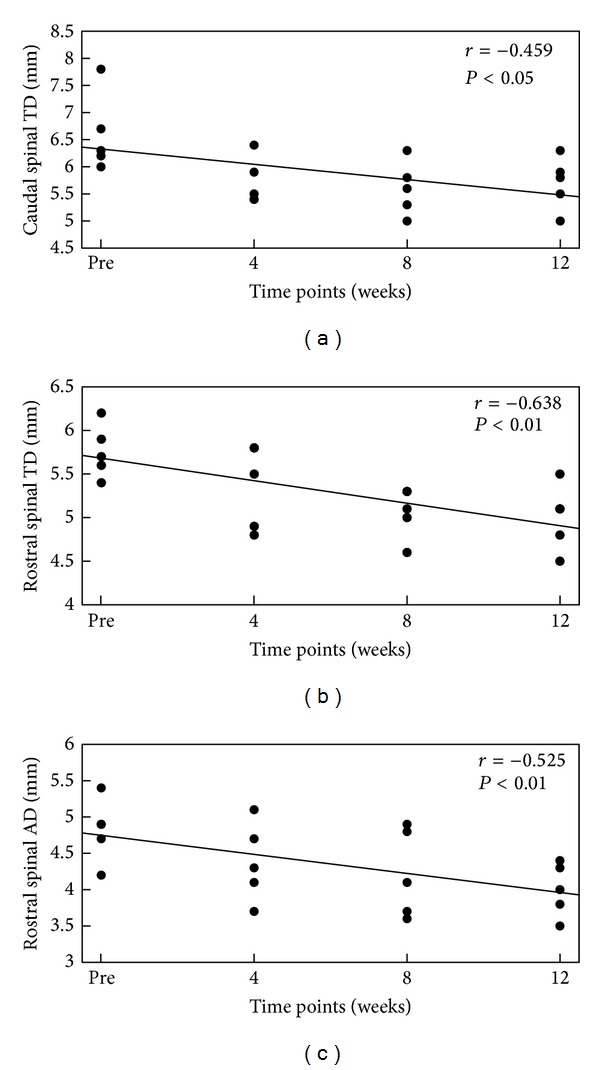
Correlations among the caudal spinal cord TD (a), rostral spinal cord TD (b), and rostral spinal cord AD (c) and different time points after the lesion. The *y*-axis in each diagram indicates the measured diameter of the spinal cord at different locations, and the *x*-axis is the time point. The straight line in the figure is the linear regression line of the matching data. Several data points with the same value overlapped in the figure. The values are reported in Supplementary Table 1 (see Supplementary Table 1 in Supplementary Material available online at http://dx.doi.org/10.1155/2013/753061).

Caudal TD was significantly correlated with the time points (*r* = −0.459, *P* = 0.021), rostral TD and time points (*r* = −0.638, *P* = 0.001), and rostral AD and time points (*r* = −0.525, *P* = 0.009) (see Figure 2). Correlation was not found between the TD and AD of the cervical cord and the time points.

### 4.2. fMRI Activation Sites of S1

**Figure 3:**
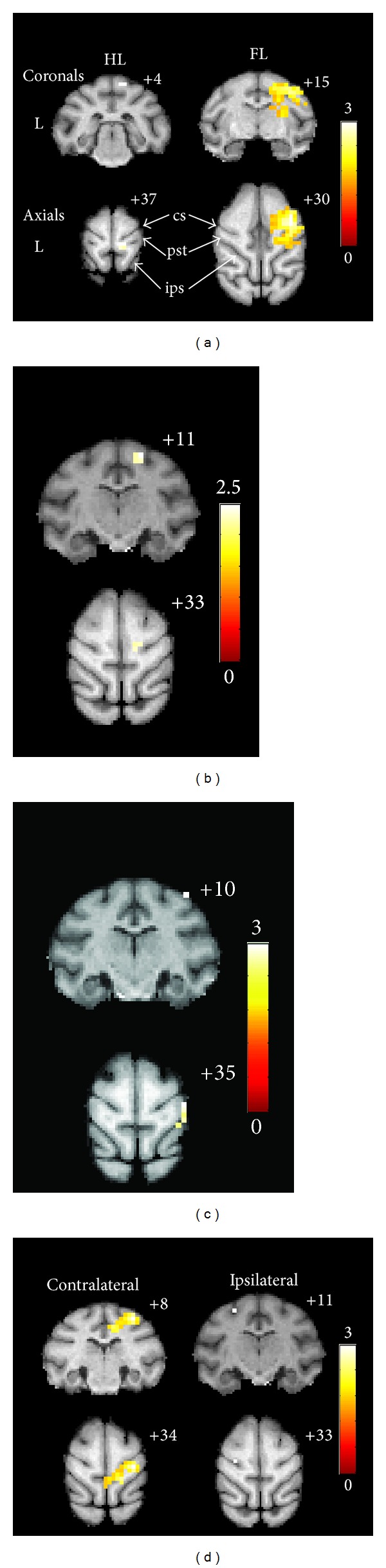
Stimulation-induced posterior central gyrus activation was superimposed on the standard monkey brain atlas: for a representative subject, the enhanced BOLD signal intensity occurred before SCI (a) and 4 weeks (b), 8 weeks (c), and 12 weeks (d) after injury through innocuous temperature stimulation to the left limbs. Color bars indicate the T-scores. (a) Stimulation of the left lower limb (HL) and left upper limb (FL) activated different areas of the c-S1. The lower limb activation region shows a medial tendency, whereas the upper limb activation region shows a lateral tendency. ((b) and (c)) Changes in the positions of the S1 activation clusters after stimulation of the left upper limb. (d) Activation response of bilateral S1 at 12 weeks after the operation. The MNI coordinates of the slices are marked on the upper right corner of each diagram. L represents the left side. Central sulcus (cs), posterior central gyrus (pst), and intraparietal sulcus (ips) were identified using arrows.

Stimulation to the left lower limb during the healthy period of monkeys presented normal activation in c-S1 (Figure 3(a)). The cortical activation was positioned in the medial S1 area of the posterior central gyrus, which is the expected sensory projection area of the lower limb. The mean X, Y, and Z coordinates of the active cluster peak are 5.2 ± 1.8, 5.2 ± 1.8, and 37.6 ± 3.0, respectively.

During the healthy period, the monkeys showed normal activation in c-S1 when the left upper limb was treated with cold stimulation (Figure 3(a)). Peak cortical activation was determined at the lateral S1 area of the posterior central gyrus, which is the expected sensory projection area of the upper limb [30]. The mean X, Y, and Z coordinates were 16.8 ± 3.0, 14.4 ± 1.7, and 32.4 ± 3.3; the mean SD between the anatomical landmarks and the MNI coordinates was 21.3 mm ± 3.5 mm; and the mean SA between the anatomical landmark and the MNI coordinates was 66.2° ± 8.8°. Significant activation of i-S1 was not observed.

Four weeks after SCI, stimulation of the left upper limb presented c-S1 activation (Figure 3(b)). The mean X, Y, and Z coordinates of the activation peak were 8.4 ± 5.5, 9.2 ± 2.3, and 34.8 ± 3.0, respectively; the mean SD between the anatomical landmark and the MNI coordinates was 11.7 mm ± 5.7 mm; and the mean SA between the anatomical landmark and the MNI coordinates is 52.6° ± 19.0°. Compared with the stimulation during the healthy period, the position of the active cluster peak was transferred toward the medial (*P* = 0.046) and posterior direction (*P* = 0.021). The activated SD was remarkably smaller than that during the healthy period (*P* = 0.015), whereas the SA showed no significant difference by comparison (*P* > 0.05).

Eight weeks after SCI, stimulation occurred at the c-S1 region (Figure 3(c)). The mean X, Y, and Z coordinates of the activation peak are 11.2 ± 5.4, 8.0 ± 2.4, and 34.8 ± 1.8, respectively; the mean SD between the anatomical landmark and the MNI coordinates is 13.6 mm ± 4.7 mm; and the mean SA between the anatomical landmark and MNI coordinates is 58.1° ± 15.9°. Compared with the stimulation during the healthy period, the position of the active cluster peak was obviously transferred toward the posterior direction (*P* = 0.004). However, the SD and the SA did not significantly differ from those during the healthy stage and those at 4 weeks after SCI.

Twelve weeks after SCI, remarkable c-S1 activation still occurred under stimulation of the left upper limb (Figure 3(d)). The mean X, Y, and Z coordinates of the activation center were 5.6 ± 2.6, 4.8 ± 3.0, and 38.0 ± 1.4; the mean SD between the anatomical landmark and the MNI coordinates was 7.1 mm ± 2.2 mm; and the mean SA between the anatomical landmark and the MNI coordinates was 60.3° ± 22.5°. Compared with the stimulation during the healthy period, the position of the active cluster peak shifted medially (*P* = 0.005), posteriorly (*P* = 0.000), and superiorly (*P* = 0.017). The activated SD was remarkably smaller than that in healthy period (*P* = 0.000) but showed no statistical difference compared with that at four weeks and at eight weeks after SCI. The SA value did not significantly differ from those of the other periods. Meanwhile, the stimulation significantly activated i-S1. The mean X, Y, and Z coordinates of the activation center were −15.6 ± 7.5, 11.2 ± 4.1, and 34.4 ± 3.6; the mean SD between the anatomical landmark and the MNI coordinates was 18.3 mm ± 8.7 mm; and the mean SA between the anatomical landmark and the MNI coordinates was 69.8° ± 5.4°. The ipsilateral cluster SD, the contralateral cluster SD, and the healthy contralateral SD did not exhibit significant differences (*P* > 0.05), similar to SA (*P* > 0.05).

At each time point after the SCI, the same area of the left lower limb of monkeys was stimulated but did not elicit activation of the c-S1 in the typical area of the lower limb.

### 4.3. Correlation between S1 Cortical Reorganization and Other Variables

**Figure 4:**
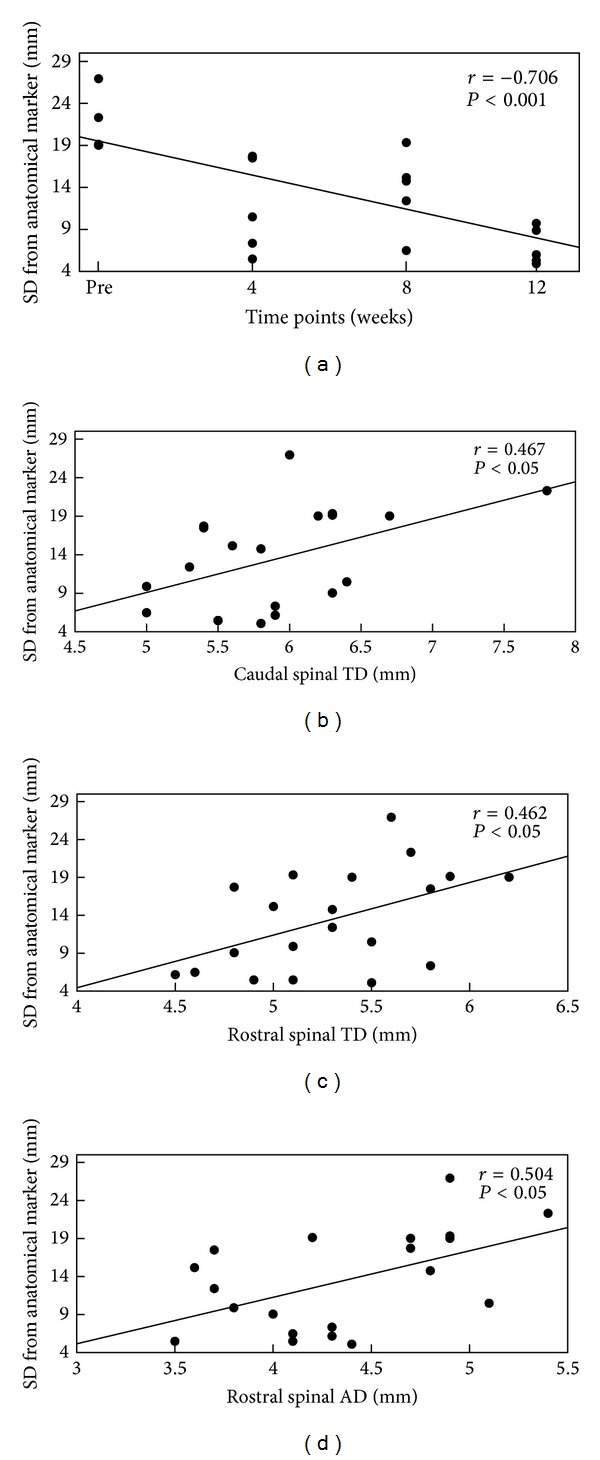
Relationship between S1 cortical reorganization and time after lesion (a), caudal spinal cord TD (b), and rostral spinal cord TD (c) and AD (d): the linear correlation diagram of the average spatial distance (SD) between the maximum activation voxel of the posterior central gyrus opposite to the anatomic landmarks in all monkeys and the spinal cord diameter (along the *x*-axis). Smaller SD indicates a higher degree of S1 reorganization.

We observed significant correlations between S1 activation SD and time points (*r* = −0.706, *P* = 0.000), between the cortical activation SD and the caudal spinal cord TD of the injury region (*r* = 0.467, *P* = 0.019), between the cortical activation SD and the rostral spinal cord TD in the injury region (*r* = 0.462, *P* = 0.020), and between the cortical activation SD and the rostral spinal cord AD in the injured region (*r* = 0.504, *P* = 0.012) (Figure 4). The cortical activation SD was not correlated with the diameter of the cervical cord. In addition, S1 activation SA was not significantly correlated with any of the variables.

## 5. Discussion 

We determined the changes in spinal cord atrophy and S1 reorganization in monkeys after unilateral thoracic SCI at different time points. Stimulation of the upper limb gradually shifted the cortical response to the posteromedial direction after the lesion. Obvious ipsilateral cortex activation was observed 12 weeks after SCI. To our knowledge, this is the first longitudinal study on the dynamic S1 reorganization of the upper limbs of adult primates after incomplete thoracic cord injury.

After SCI, the ascending and descending fiber tracts were interrupted and the axon of the injured area was damaged, which caused subsequent disintegration towards the rostral and the caudal directions; that is, secondary degeneration occurred [31]. Freund et al. [14] demonstrated that different degrees of cervical cord injury reduce the average area of the cervical cord. However, they did not specifically measure the rostral and the caudal spinal cord diameters of the areas adjacent to the injury. According to our findings, following the unilateral thoracic spinal cord injury, the TD and AD of the rostral and the aspects of the caudal spinal cord demonstrated a tendency to shrink, but only the rostral spinal cord TD significantly differed from that under healthy conditions. At 12 weeks after the lesion, the rostral spinal cord TD 2 cm from the injured region was significantly decreased. In addition, the rostral spinal cord TD was significantly related to the time points after the lesion. The lateral atrophy of the spinal cord may have contributed to this condition; that is, the corticospinal tract developed neurodegenerative changes [13, 14], including axonal degeneration, demyelination, loss of neurons, and death [32]. Caudal spinal cord TD and rostral spinal cord AD of the injured region decreased with time after the lesion but did not significantly differ from those during the healthy period. This finding may be partly caused by the unilateral spinal cord injury in the animal models, which connected healthy neurons with injured neurons to compensate for the functional loss. Furthermore, our findings demonstrate that the TD and AD of the cervical cord did not change significantly at any time point during the experiment. These results differed from those by Lundell et al. [7]. Positional differences in the SCI may have caused this discrepancy between the two studies.

As expected, the healthy animals only reacted to the stimulation with the contralateral somatosensory cortical mapping area, which was consistent with the conventional sensory model. The representatives of the upper limb were closer to the anterior and lateral sides than the representatives of the lower limb [33, 34]. Correspondingly, after surgical SCI, stimulation of the lower limb did not significantly activate the somatosensory cortex. Stimulation of the upper limb shifted the activated cortex region posteromedially. This finding is consistent with the transfer of S1 representations of the upper limb to S1 areas that generally dominate the lower body [18, 29]. The posteromedial shift in the activation of the upper limb was enhanced over time. The distance between the position of the activation cluster peak and the anatomical landmark was negatively correlated with the time points after the lesion.

Cortical reorganization after peripheral afferent nerve damage [9, 35] and central nervous system injury [7, 18, 36, 37] has been widely acknowledged and reported. However, the mechanism of cortical reorganization remains unclear. Some researchers believe that cortical reorganization results from the activation of existing dormant synapses [38], and others consider it as the result of physical changes of brain anatomy caused by obstructed afferent nerves [39, 40]. In our study, compared with the healthy period, the cortical activation cluster significantly shifted medially 4 weeks after SCI and shifted posteromedially and superiorly 12 weeks after the lesion. Changes in the direction of the displacement of the cortical activation clusters are unlikely to be induced by the activation of the existing dormant synapses because the existing dormant synapses have already been swiftly activated in the early stage of injury [38].

The findings 12 weeks after SCI revealed the activation of the ipsilateral S1. The activated cluster peak was positioned between the cortical representatives of the lower limb and the upper limb. Previous studies showed anatomical changes in the bilateral cortex after complete SCI [41] and activated the bilateral cortex because of its involvement in task processing [19, 29]. Lundell et al. [7] demonstrated that motor task in the lower limb induced large-scale activation of the bilateral cortex in incomplete chronic SCI patients. Nishimura et al. [42] also illustrated the motor-induced activation of the bilateral motor cortex among monkeys with unilateral cervical cord corticospinal tract injury. Although we observed bilateral cortex activation, our study differs from previous studies because it focuses on sensory information. Most significantly, the conduction pathway of the upper limb was not directly injured. Thus, the underlying mechanism of bilateral somatosensory cortical activation requires further exploration.

The shorter distance between the cortical activation cluster peak and the anatomical landmark over time indicates that the degree of cortical reorganization increases over time. This finding is consistent with previous studies [18, 19] and corroborates the positive correlation between the degree of cortical reorganization and the time after incomplete SCI. Similar to the findings by Lundell et al. [7], we observed a negative correlation between the degree of cortical reorganization and rostral spinal cord TD in the injured region. Considering that approximately 30% of the corticospinal tract originates from the S1 of the posterior central gyrus [43], corticospinal tract injury may induce the establishment of a connection between the sensory system and the motor system [44], thereby adjusting the cortical plasticity. In addition, our findings also indicate a positive correlation between the degree of cortical reorganization and the rostral spinal cord AD atrophy because the ascending sensory pathways toward S1 are mainly located in the white matter of the ventral and dorsal spinal cord. The anteroposterior atrophy of the spinal cord reflects the injury to the ascending sensory pathways, which causes the degradation and loss of sensory function. However, in this study, the spinal cord, including the sensory pathway of the upper limb, was not directly injured and the diameter of the cervical cord was unchanged. Thus, cortical reorganization may not be conducted by the changes in the sensory pathway of the upper limb. The relationship between cortical reorganization and rostral spinal cord AD of the injury region may be interpreted as follows: first, the injury weakens the inhibition of *γ*-aminobutyric acid (GABA), which leads to disinhibition excitement [45, 46]; second, as the neurons are transected and degraded, the cytoactivity is eliminated, which triggers the atrophy of the S1 neurons [14, 47] and compensates by adjusting the central mechanism; third, the severe loss of sensory feedback promotes the growth of new axonal and dendritic sprouting, which connect different sensory representative areas [48, 49]. Notably, spinal cord diameter atrophy predicted the shift in cortical activation induced by sensory tasks to the mapping representatives of the lower limb, which indicates that more severe spinal cord atrophy will induce more intensive cortical reorganization.

The refined underlying mechanism of cerebral cortical reorganization was not clarified. Following SCI, the spinal cord exhibited changes in structure and function to cope with the disruption in the ascending and descending nerve fiber tracts [23, 50]. The changes at the spinal level likely contributed to cortical reorganization. This study determined the relationship between spinal cord diameter atrophy and cortical reorganization, but we failed to quantify the functional reorganization at the spinal level, and its effect on cortical reorganization should be further investigated. Moreover, because we failed to find a proper assessment system, somatosensory cortical reorganization induced by changes in the usage patterns of upper limbs cannot be excluded although we did not observe the excessive use of the upper limbs among the experimental monkeys after SCI.

## 6. Conclusions

We examined time-associated spinal cord atrophy, function reorganization in S1, and bilateral cortical activation after incomplete SCI. The fMRI results verify the correlation among the displacement of cortical activation, the reduction in spinal cord diameter, and the time points after SCI. Based on these findings, cerebral cortical reorganization after SCI includes the bilateral somatosensory cortex region and spinal cord diameter may be a useful marker for rapidly assessing the degree of SCI-induced cortical reorganization.

## Supplementary Material

Supplementary Table 1 Raw data of the spinal cord diameters and the activated cluster's spatial distance from the anatomical marker (mm).

## Figures and Tables

**Table 1:** Average of spinal cord diameters at all time points. All data are given as means ± standard deviation (mm).

	Health	4 w after injury	8 w after injury	12 w after injury
Caudal TD	6.60 ± 0.71	5.72 ± 0.43	5.60 ± 0.49	5.70 ± 0.48
Caudal AD	5.26 ± 0.59	4.82 ± 0.79	4.90 ± 0.67	4.74 ± 0.77
Rostral TD	5.76 ± 0.30	5.36 ± 0.48	5.06 ± 0.29	5.00 ± 0.37*
Rostral AD	4.82 ± 0.43	4.38 ± 0.54	4.22 ± 0.61	4.00 ± 0.37
Cervical TD	5.78 ± 0.24	5.52 ± 0.165	5.48 ± 0.19	5.62 ± 0.11
Cervical AD	4.44 ± 0.18	4.36 ± 0.21	4.50 ± 0.19	4.20 ± 0.21

w: week; TD: transverse diameter; AD: anteroposterior diameter; *P < 0.05.
