# Supplementary material for: Atrophy and Primary Somatosensory Cortical Reorganization after Unilateral Thoracic Spinal Cord Injury: A Longitudinal Functional Magnetic Resonance Imaging Study
Source: Biomed Res Int. 2013 Dec 29;2013:753061. doi: 10.1155/2013/753061 (PMC3891744; doi:10.1155/2013/753061)
Supplement: Supplementary file 1 — Supplementary Table 1 Raw data of the spinal cord diameters and the activated cluster's spatial distance from the anatomical marker (mm). [file 753061.f1.doc]

**Supplemental Information**

**Supplementary Table 1** Raw data of the spinal cord diameters and the activated cluster’s spatial distance from the anatomical marker (mm).

| Timepoint(w) | Number | Caudal TD | Caudal AD | Rostral TD | Rostral AD | SD |
| --- | --- | --- | --- | --- | --- | --- |
| pre | Monkey 1 | 7.80 | 6.30 | 5.70 | 5.40 | 22.315900 |
| pre | Monkey 2 | 6.00 | 5.10 | 5.60 | 4.90 | 26.944400 |
| pre | Monkey 3 | 6.30 | 4.80 | 5.90 | 4.20 | 19.131100 |
| pre | Monkey 4 | 6.70 | 5.10 | 6.20 | 4.70 | 19.026300 |
| pre | Monkey 5 | 6.20 | 5.00 | 5.40 | 4.90 | 19.026300 |
| 4 | Monkey 1 | 6.40 | 6.20 | 5.50 | 5.10 | 10.488100 |
| 4 | Monkey 2 | 5.40 | 4.30 | 4.80 | 4.70 | 17.720000 |
| 4 | Monkey 3 | 5.40 | 4.40 | 5.80 | 3.70 | 17.492900 |
| 4 | Monkey 4 | 5.90 | 4.50 | 5.80 | 4.30 | 7.348500 |
| 4 | Monkey 5 | 5.50 | 4.70 | 4.90 | 4.10 | 5.477200 |
| 8 | Monkey 1 | 6.30 | 6.10 | 5.10 | 4.90 | 19.339100 |
| 8 | Monkey 2 | 5.60 | 4.60 | 5.00 | 3.60 | 15.165800 |
| 8 | Monkey 3 | 5.30 | 4.60 | 5.30 | 3.70 | 12.409700 |
| 8 | Monkey 4 | 5.80 | 4.70 | 5.30 | 4.80 | 14.764800 |
| 8 | Monkey 5 | 5.00 | 4.50 | 4.60 | 4.10 | 6.480700 |
| 12 | Monkey 1 | 6.30 | 6.00 | 4.80 | 4.00 | 9.055400 |
| 12 | Monkey 2 | 5.90 | 4.80 | 4.50 | 4.30 | 6.164400 |
| 12 | Monkey 3 | 5.50 | 4.30 | 5.10 | 3.50 | 5.477200 |
| 12 | Monkey 4 | 5.80 | 4.60 | 5.50 | 4.40 | 5.099000 |
| 12 | Monkey 5 | 5.00 | 4.00 | 5.10 | 3.80 | 9.899500 |

W, week; TD, trans diameter; AD, anteroposterior diameter; SD, spatial distance.
